# Enhanced Inflammation is a Marker for Risk of Post-Infarct Ventricular Dysfunction and Heart Failure

**DOI:** 10.3390/ijms21030807

**Published:** 2020-01-26

**Authors:** Iwona Świątkiewicz, Przemysław Magielski, Jacek Kubica, Adena Zadourian, Anthony N. DeMaria, Pam R. Taub

**Affiliations:** 1 Department of Cardiology and Internal Medicine, Collegium Medicum, Nicolaus Copernicus University, Marii Skłodowskiej-Curie 9, 85-094 Bydgoszcz, Poland; 2 Division of Cardiovascular Medicine, University of California San Diego, 9300 Campus Point Drive MC 7410, La Jolla, CA 92037, USA

**Keywords:** acute myocardial infarction, left ventricular function, heart failure, echocardiography, inflammation, C-reactive protein

## Abstract

Acute ST-segment elevation myocardial infarction (STEMI) activates inflammation that can contribute to left ventricular systolic dysfunction (LVSD) and heart failure (HF). The objective of this study was to examine whether high-sensitivity C-reactive protein (CRP) concentration is predictive of long-term post-infarct LVSD and HF. In 204 patients with a first STEMI, CRP was measured at hospital admission, 24 h (CRP_24_), discharge (CRP_DC_), and 1 month after discharge (CRP_1M_). LVSD at 6 months after discharge (LVSD_6M_) and hospitalization for HF in long-term multi-year follow-up were prospectively evaluated. LVSD_6M_ occurred in 17.6% of patients. HF hospitalization within a median follow-up of 5.6 years occurred in 45.7% of patients with LVSD_6M_ vs. 4.9% without LVSD_6M_ (*p* < 0.0001). Compared to patients without LVSD_6M_, the patients with LVSD_6M_ had higher CRP_24_ and CRP_DC_ and persistent CRP_1M_ ≥ 2 mg/L. CRP levels were also higher in patients in whom LVSD persisted at 6 months (51% of all patients who had LVSD at discharge upon index STEMI) vs. patients in whom LVSD resolved. In multivariable analysis, CRP_24_ ≥ 19.67 mg/L improved the prediction of LVSD_6M_ with an increased odds ratio of 1.47 (*p* < 0.01). Patients with LVSD_6M_ who developed HF had the highest CRP during index STEMI. Elevated CRP concentration during STEMI can serve as a synergistic marker for risk of long-term LVSD and HF.

## 1. Introduction

Post-infarct left ventricular systolic dysfunction (LVSD), which is defined as reduced left ventricular ejection fraction (LVEF), occurs frequently and is associated with unfavorable long-term outcomes including heart failure (HF) and increased cardiovascular mortality [1,2,3,4,5,6,7,8]. Post-infarct LVEF ≤ 40% indicates a high risk of adverse clinical outcomes and guides therapy in patients with ST-segment elevation myocardial infarction (STEMI) treated with percutaneous coronary intervention (PCI) [9,10].

Local and systemic inflammation plays an important role in the pathophysiology of post-infarct left ventricular damage and repair [11,12,13]. Inflammatory activation associated with myocardial infarction is essential for myocardial healing and cardiac function because of complement activation, cytokine and chemokine upregulation, leukocyte and macrophage recruitment, and initiation of fibrosis [11]. However, overactive or prolonged inflammatory response can lead to further cardiac damage, as well as long-term LVSD and HF [11,12,13].

C-reactive protein (CRP) is an acute-phase protein with prognostic significance in primary and secondary prevention [14,15,16,17,18,19,20,21,22,23,24]. CRP is a reliable biomarker of inflammation due to its long half-time, standardized laboratory assays, and ability to reflect inflammatory activity involved in myocardial damage [12,13,14,25,26]. At present, the potential value of CRP for the prediction of long-term LVSD and HF in patients with STEMI undergoing PCI and guideline-based therapies has not been definitely assessed [14,27]. Previous studies have been limited by heterogenous populations with acute coronary syndromes that were frequently untreated with PCI, small sample size, a lack of multiple high-sensitivity CRP measurements, absence of neurohormonal activation assessment, lack of long-term monitoring of LVEF and HF, and omission of long-term LVSD and HF as clinical endpoints [14,16,17,18,21,22,23,24,25,26,27,28,29,30,31,32,33,34].

The purpose of this study was to assess the value of high-sensitivity CRP in a homogenous population of patients with first STEMI undergoing primary PCI and guideline-based therapies for predicting the risk of: (i) LVSD at 6 months after hospital discharge (LVSD_6M_), which was the primary study endpoint; and (ii) the need for hospitalization for HF in patients with LVSD_6M_ in long-term multi-year follow-up, which was the secondary study endpoint. This secondary endpoint was chosen because hospitalization for HF is associated with subsequent increase in risk of mortality [8]. We performed a single-center prospective cohort study with rigorous selection criteria, adequate sample size, and long-term follow-up data based on multiple assessment time-points: baseline, 24 h, and discharge during index hospitalization for STEMI, 1 month and 6 months after discharge, and long-term multi-year follow-up. Serial measurements at multiple time-points of high-sensitivity CRP concentration were made to determine the optimal time point that is the strongest predictor of clinical outcomes. These time points included hospital admission (CRP_AD_), 24 h after admission (CRP_24_), discharge (CRP_DC_), and 1 month after discharge (CRP_1M_) post-index hospitalization for STEMI. Echocardiography was performed at discharge upon index hospitalization for STEMI and after 6 months to assess LVEF. LVSD_6M_ was defined as LVEF ≤ 40% at 6 months after discharge post-STEMI [1,2,4,9]. HF hospitalization in the long-term follow-up was defined as post-index STEMI readmission due to new or increasing symptoms and signs of HF in combination with a change in treatment to improve HF [8]. Neurohormonal activation and hemodynamic stress was assessed using B-type natriuretic peptide (BNP) plasma concentration.

## 2. Results

During the study period (from December 2005 to December 2008), 4311 patients with acute myocardial infarction were treated invasively in our center. Out of 2730 patients with STEMI who were screened to determine their eligibility for participation in this study, initially 217 consecutive STEMI patients satisfied inclusion criteria and gave informed consent for participation in the study at the admission to hospital for STEMI. During the index hospitalization for STEMI, 13 patients were excluded from the study due to false diagnosis of STEMI (3 patients), insufficient quality of echocardiographic images for quantitative analysis (4 patients), expected difficulties in cooperation due to alcohol abuse (1 patient) or dementia (1 patient), cardiogenic shock requiring dopamine therapy (1 patient), acute pharyngitis (1 patient), abdominal aneurysm rupture (1 patient), and withdrawal of consent (1 patient). As a result, 204 (156 men and 48 women) patients were enrolled in this study. Out of these 204 patients, 3 patients died during the first 6 months and 2 patients did not attend the 6-month visit. Therefore, 199 patients had 6-month echocardiography and were analyzed for LVSD_6M_ (defined as LVEF ≤ 40%) that was the primary study endpoint. Out of these 199 patients, 35 patients who exhibited LVSD_6M_ were subject to long-term follow-up for HF hospitalization, which was the secondary study endpoint, with a median observation period of ~5.6 years (4.9–6.3 years). After 6 months, two patients died, and these two patients exhibited LVSD_6M_. All patients received standard treatment following current guidelines on the management of acute STEMI developed by the European Society of Cardiology, as described in the Materials and Methods section (Section 4.2.) [35,36]. As a result, as shown in Table 1, all patients received dual antiplatelet therapy (clopidogrel up to 1 year and aspirin, each at 75 mg q.d.) and high-dose statin, and almost all of them received beta blocker (99% of patients) and angiotensin-converting-enzyme inhibitor (99.5%). Primary PCI resulted in a complete restoration of epicardial blood flow in the infarct-related artery in the majority of patients (93%) and successful myocardial reperfusion in almost half of patients (46.2%).

### 2.1. Study Endpoints

**Figure 1:**
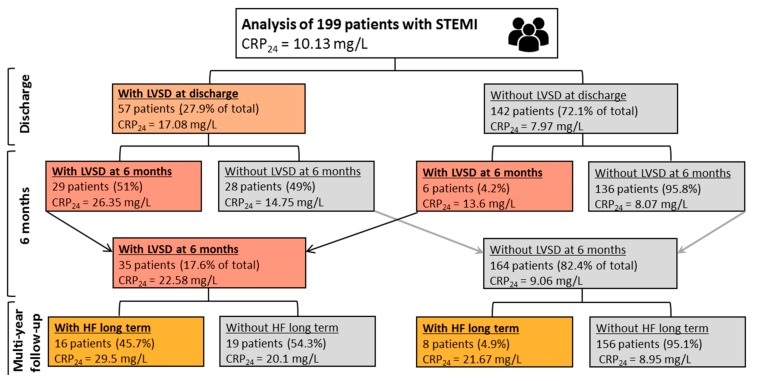
Occurrence of left ventricular systolic dysfunction (LVSD) at discharge and 6 months after discharge, and hospitalization for heart failure (HF) in long-term follow-up, with corresponding median concentration of C-reactive protein 24h after admission (CRP_24_) for ST-segment elevation myocardial infarction (STEMI).

As shown in Figure 1, 57 patients (27.9% of total) had LVSD at discharge (LVSD_DC_), 29 of whom (51%) had persistent LVSD_6M_. Of 142 patients without LVSD_DC_, only 6 patients (4.2%) developed LVSD_6M_. At 6 months, 35 patients (17.6% of the total) had LVSD_6M_. The comparison of various characteristics for the groups of patients with and without LVSD_6M_ is given in Table 1 and Table 2.

As shown in Figure 1, HF hospitalization in long-term follow-up occurred more often in patients with LVSD_6M_ (45.7%) than in those without LVSD_6M_ (4.9%; *p* < 0.0001). Importantly, HF hospitalization occurred in 29.8% of patients with LVSD_DC_ and in 4.8% of patients without LVSD_DC_.

The patients with LVSD_6M_ had more frequent anterior STEMI and mild HF prior to STEMI, and were more likely to have diabetes mellitus, hypertension, higher body mass index, and unfavorable pre-PCI angiographic indices than patients without LVSD_6M_ (Table 1 and Table 2). In the majority of patients, PCI resulted in a complete restoration of epicardial blood flow in the infarct-related artery, however, with less reperfusion success in patients with LVSD_6M_ (Table 2). All patients with LVSD_6M_ had intracoronary stents implanted and received all guideline-recommended medications as described in the Materials and Methods section (Table 1 and Table 2). The same treatment was administered in nearly all patients without LVSD_6M_. Importantly, there were no significant differences in the percentage of patients receiving treatment from the groups with and without LVSD_6M_ (Table 1 and Table 2).

**Figure 2:**
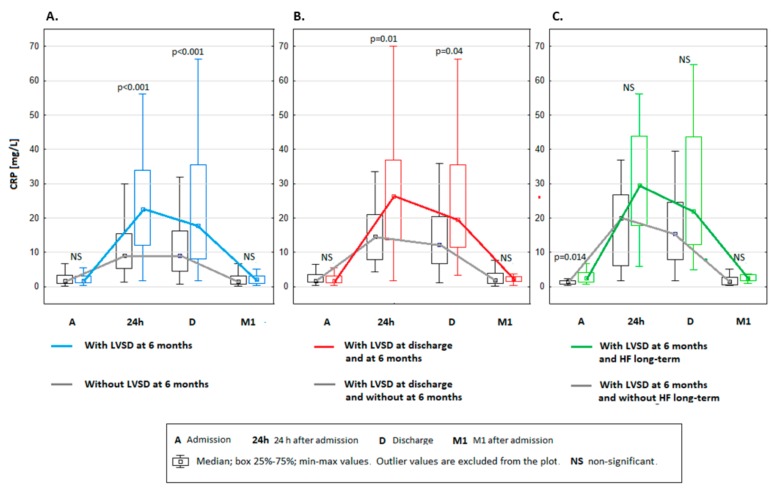
Comparison of C-reactive protein (CRP) median concentration during hospitalization for ST-segment elevation myocardial infarction and at 1 month after discharge between different groups of patients: (**A**) with (*n* = 35 patients) and without (*n* = 164) left ventricular systolic dysfunction (LVSD) 6 months after discharge; (**B**) with LVSD at discharge (*n* = 57) and who either exhibited LVSD 6 months after discharge (*n* = 29) or did not exhibit LVSD after 6 months (*n* = 28); (**C**) with LVSD 6 months after discharge (*n* = 35) who either had hospitalization for heart failure (HF) (*n* = 16) or did not have hospitalization for HF (*n* = 19) in long-term follow-up.

**Figure 3:**
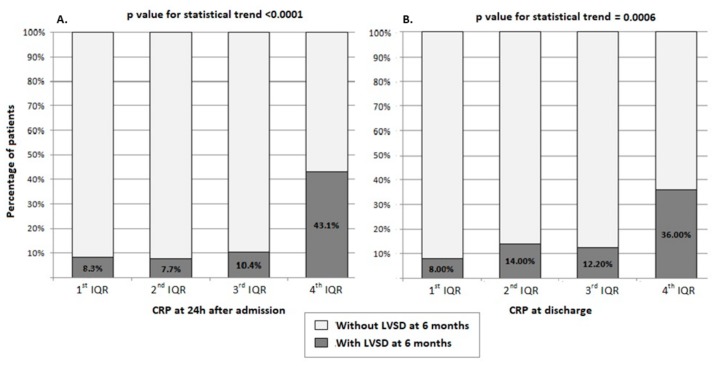
Prevalence of left ventricular systolic dysfunction (LVSD) 6 months after discharge post-ST-segment elevation myocardial infarction based on quartiles of C-reactive protein (CRP) concentration: (**A**) 24 h after admission (*n* = 4 patients in 1st quartile, *n* = 4 in 2nd quartile, *n* = 5 in 3rd quartile, *n* = 22 in 4th quartile); (**B**) at discharge (*n* = 4 in 1st quartile, *n* = 7 in 2nd quartile, *n* = 6 in 3rd quartile, *n* = 18 in 4th quartile).

The patients with LVSD_6M_ had higher myocardial necrosis indices and BNP levels, and lower LVEF during index hospitalization for STEMI than patients without LVSD_6M_ (Table 1 and Table 2). CRP concentration increased during the first 24 h of hospitalization and remained elevated up to discharge (Table 1). Figure 2A–C compares the CRP concentration during hospitalization for STEMI and at 1 month after discharge between different groups of patients. CRP remained elevated at discharge in patients with LVSD_6M_ to a greater extent than in those without LVSD_6M_, and hence CRP_24_ and CRP_DC_ were significantly higher in patients with LVSD_6M_ than without LVSD_6M_ (Figure 2A). The patients with LVSD_6M_ had persistent elevation in CRP at 1 month after discharge with median CRP_1M_ ≥ 2 mg/L in contrast to those without LVSD_6M_. The analysis of CRP levels by quartiles revealed that higher CRP_24_ and CRP_DC_ resulted in greater prevalence of LVSD_6M_ (Figure 3). LVSD_6M_ occurred in 43.1% of patients with CRP_24_ within the 4^th^ quartile (cut-off value ≥19.64 mg/L) vs. 8.3% within the 1^st^ quartile (≤5.62 mg/L) (Figure 3A), and in 36% of patients with CRP_DC_ within the 4^th^ quartile (≥17.65 mg/L) vs. 8% within the 1^st^ quartile (≤4.91 mg/L) (Figure 3B).

Of the 57 patients who had LVSD_DC_, CRP_24_ and CRP_DC_ were significantly higher and were accompanied with persistent median CRP_1M_ ≥ 2 mg/L in 29 patients (51% of all patients with LVSD_DC_) in whom LVSD persisted at 6 months vs. 28 patients (49%) in whom LVSD resolved (CRP_24_ = 26.35 vs. 14.75 mg/L, *p* = 0.01; CRP_DC_ = 19.6 vs. 12.2 mg/L, *p* = 0.041). However, despite persistent CRP_1M_ ≥ 2 mg/L, CRP_1M_ was not significantly different between these two groups of patients (CRP_1M_ = 2.33 vs. 1.95 mg/L, *p* = 0.745). 

Among the groups with LVSD (Figure 1), the highest median CRP was observed in patients with LVSD at both discharge and 6 months (Figure 2B). Moderate correlations between maximal activity of creatine-kinase MB (which was used as a measure of infarct size) and CRP_24_ (*r* = 0.39; *p* < 0.001) or CRP_DC_ (*r* = 0.36; *p* < 0.001) were found.

Of the patients with LVSD_6M_, no significant differences were observed during index hospitalization for STEMI in BNP levels, myocardial necrosis indices, and LVEF between patients who developed HF long term and those who did not (Table 3). There were also no significant differences in the percentage of patients receiving guideline-based treatments from the groups with and without HF hospitalization. However, CRP_AD_ was significantly higher and CRP_24_ and CRP_DC_ were likely to be higher in patients with HF hospitalization in the long-term follow-up than those without HF (Table 3, Figure 2C). Moreover, the patients with the HF had persistently elevated CRP after discharge with median CRP_1M_ ≥ 2 mg/L in contrast to those without HF. Among all analyzed groups (Figure 1), the highest CRP median concentrations during STEMI (CRP_24_ = 29.5 mg/L) and 1 month after discharge (2.57 mg/L) were observed in patients with LVSD_6M_ who developed HF long term (Table 3, Figure 2A–C).

**Figure 4:**
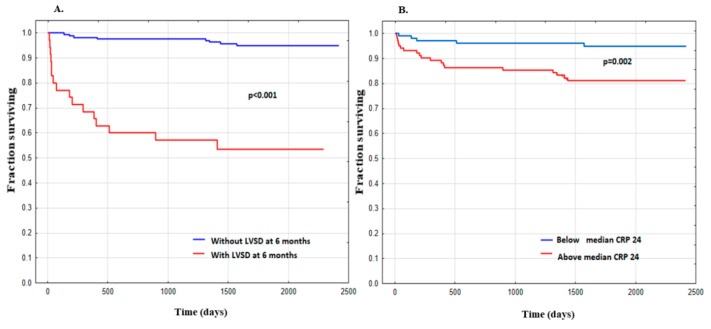
Kaplan–Meier analysis showing survival free from hospitalization for heart failure in long-term follow-up in groups of patients: (**A**) with (*n* = 35) and without (*n* = 164) left ventricular systolic dysfunction (LVSD) 6 months post-ST-segment elevation myocardial infarction; (**B**) with the concentration of CRP 24 h after admission (CRP_24_) for ST-segment elevation myocardial infarction below (*n* = 102) or above (*n* = 102) median value.

Kaplan–Meier analysis showed decreased probability of long-term survival free from HF hospitalization in patients with LVSD_6M_ compared with those without LVSD_6M_ (Figure 4A) and when CRP_24_ was higher than the median value (Figure 4B).

### 2.2. Prognostic Factors

Univariate logistic regression analysis revealed predictors of LVSD_6M_ based on baseline characteristics (Table 4). The first basic multivariable logistic regression model indicated low LVEF and high BNP concentration at discharge as independent predictors of LVSD_6M_ (Table 4). In the second multivariable model that purposely excluded LVEF at discharge (see Materials and Methods section for explanation), the infarct-related left anterior descending artery, increased BNP concentration at discharge, and high CRP_24_ identified high risk of LVSD_6M_ (Table 4). Owing to this approach, our analysis was able to reveal that CRP concentration provides a synergistic prognostic value in conjunction with the commonly used conventional risk factors such as the infarct-related left anterior descending artery and high BNP concentration.

The receiver-operator-characteristic-derived areas under the curves were 0.91 for LVEF at discharge (95% CI 0.86–0.94), 0.84 for BNP concentration at discharge (95% CI 0.78–0.88), and 0.74 for CRP_24_ (95% CI 0.67–0.80). The corresponding optimal cut-off values were 40.4% for LVEF at discharge (86% sensitivity, 82% specificity, 50% positive predictive value, 96% negative predictive value), 226.5 pg/mL for BNP concentration at discharge (77%, 85%, 53%, 95%, respectively), and 19.67 mg/L for CRP_24_ (63%, 83%, 44%, 91% respectively). Based on receiver operator characteristic analysis, the BNP predictive value for LVSD_6M_ was only marginally better than CRP (*p* = 0.054), and LVEF was better than both BNP (*p* = 0.076) and CRP (*p* < 0.001).

## 3. Discussion

Our study emphasizes the importance of inflammatory pathways in cardiovascular pathogenesis and, to our knowledge, represents the first study to investigate the role of serial high-sensitivity CRP concentrations for predicting the risk of long-term LVSD and HF in STEMI patients. We established that the intensity and duration of inflammatory activation during STEMI as manifested by elevated CRP is associated with an increased risk of long-term LVSD and subsequent HF hospitalization in multi-year follow-up for a homogeneous population receiving early successful PCI and guideline-based therapies. These results indicate that elevated CRP has a synergistic prognostic value which, along with conventional risk factors such as the infarct-related left anterior descending artery, high BNP concentration, and low LVEF, can improve the identification of high-risk patients. 

Our primary findings are: (1) LVSD_6M_ occurred in 17.6% of STEMI patients and was associated with a 9.3-fold increased risk of HF hospitalization within a median period of 5.6 years as 45.7% of patients with LVSD_6M_ required hospitalization for HF; (2) CRP_24_ and CRP_DC_ concentrations were significantly higher with persistent CRP_1M_ ≥ 2 mg/L in patients with LVSD_6M_ compared to those without LVSD_6M_; (3) of patients who had LVSD_DC_, the concentrations of CRP_24_ and CRP_DC_ were significantly higher with persistent CRP_1M_ ≥ 2 mg/L in the patients who had persistent LVSD_6M_ (51% of all patients with LVSD_DC_) compared to those patients in whom LVSD resolved; (4) elevated CRP_24_ ≥ 19.67 mg/L was associated with an increased odds ratio for LVSD_6M_ of 1.47 (*p* < 0.01) in multivariable analysis; (5) patients with LVSD_6M_ who had HF hospitalization in the long-term follow-up had the highest CRP concentrations throughout index hospitalization for STEMI and afterwards with persistent CRP_1M_ ≥ 2 mg/L.

Previous studies of the prognostic value of CRP for LVSD and HF in STEMI patients had significant limitations, such as retrospective case–control design, prior myocardial damage, LVEF assessed as continuous variable at a single early time point or not assessed at all, a single CRP measurement taken during initial hours of the hospitalization, low-sensitivity CRP assays, small heterogeneous populations, short-term observations, and different treatment strategies [16,18,21,22,23,24,25,26,27,28,29,30,31,32,33,34]. Moreover, no long-term LVSD and/or HF as a clinical endpoint was reported [18,21,22,24,25,26,30,34]. Consequently, the existing results are conflicting and provide no convincing evidence for a prognostic value of CRP [14,30,33,34]. A few studies demonstrated some association between CRP and LVEF but were subject to limitations as mentioned above, most importantly, the study populations were not consistently treated with PCI and the observations were limited to the period before hospital discharge or short-term post-discharge [21,22,24,25,26,28,29]. However, our previous observations showed that CRP_DC_ can detect early LVSD following a first STEMI treated with PCI with a greater discriminating value than BNP [29]. In some other studies a relationship between CRP and post-infarct HF was observed, but the results are limited in terms of heterogeneous populations with the different types of acute coronary syndromes, lack of therapy with PCI in all patients, lack of LVEF assessment, omission of HF as a single clinical endpoint, and observational periods no longer than 28 months [16,18,23,31,32].

CRP is released as a response to stimulation by interleukin (IL)-6 produced in ischemic zone, thus measuring the intensity of inflammation [11,12,13,14,25,26]. A correlation between CRP, infarct size, and markers of myocardial necrosis have been reported [25,26]. In our study, patients with the most adverse outcome (i.e., long-term LVSD and HF hospitalization) had the most impaired left ventricular regional systolic function, the highest myocardial necrosis indices, and also had the highest CRP concentrations during STEMI, which support the role of CRP as a marker of inflammation intensity in infarcted areas. In addition, CRP may be stimulated by microvasculature obstruction following STEMI as defined by an impairment of flow in damaged capillaries in the central infarct zone [25,26]. In our study, patients with LVSD_6M_ and high CRP exhibited sub-optimal microvascular flow post-PCI.

Impaired regulation of inflammatory response during myocardial infarction contributes to prolongation or expansion of inflammation and may be involved in extending myocardial injury, resulting in left ventricular dysfunction [11,12,13,14,37,38]. Owing to pronecrotic and proatherogenic features, CRP may directly contribute to an unfavorable course of inflammatory activation [11,12,13,14,37]. In several experimental studies, CRP has been implicated in various adverse processes extending myocardial injury, such as complement deposition, apoptosis, macrophage infiltration, expression of chemokines and cytokines, phagocytic activity, production of reactive oxygen and nitric oxide, suppression of angiogenesis, renin-angiotensin system activation, and inhibition of endothelial progenitor cells [11,12,13,14,37]. In experimental models of myocardial infarction, an overexpression of CRP demonstrated greater left ventricular dysfunction, while an inhibition of exogenous CRP with a specific antagonist or apheresis decreased infarct size [11,37].

The results of our study suggest that CRP may be a mediator of inflammatory reaction during STEMI. We observed that LVSD persisted at 6 months in patients with LVSD_DC_ who had higher CRP during index hospitalization for STEMI and persistent CRP elevation 1 month after discharge. In addition, the patients with LVSD_6M_ who required HF hospitalization in multi-year follow-up had the highest CRP concentration during index hospitalization for STEMI. This was observed despite the fact that the myocardial necrosis indices and BNP levels during index STEMI, and LVEF both at discharge and at 6 months post-index hospitalization for STEMI in patients requiring HF hospitalization, were not significantly different compared to those without HF. These results are consistent with severe inflammatory reaction in patients with relatively greater increase of CRP compared to creatine kinase level, indicating an extensive infarct expansion, which was predictive of post-infarct cardiac rupture and ventricular aneurysm [11]. This is supported by our observation that, in contrast to CRP, myocardial necrosis indices were not found to be predictors of LVSD_6M_.

It is also known that slightly elevated CRP (≥2 mg/L) can detect low-grade inflammation and is useful for determining cardiovascular risk [15,16]. Thus, CRP ≥ 2 mg/L 1 month post-index hospitalization for STEMI in our patients with the most adverse outcome (i.e., LVSD_6M_ alone or also with HF hospitalization long term) is consistent with a persistent inflammatory response post-STEMI. Recent results from the CANTOS study showed that anti-inflammatory therapy with canakinumab, a therapeutic monoclonal antibody targeting IL-1β, resulted in reduced rate of recurrent atherosclerotic cardiovascular events in post-infarct patients with CRP ≥ 2 mg/L, especially in patients with greater CRP reduction, even in the absence of lipid lowering [39,40]. The findings of small prespecified secondary analysis of CANTOS may also indicate a positive impact of canakinumab on long-term LVEF in patients with post-infarct systolic HF [41]. CANTOS also provided proof of concept evidence in humans that modulation of the IL-6 signaling pathway—at least with canakinumab—is associated with reduced cardiovascular event rates [42]. Although the CIRT results found that anti-inflammatory treatment with low-dose methotrexate did not result in fewer cardiovascular events in post-infarct patients, the drug did not reduce levels of CRP [43]. 

Our study revealed a relationship between the intensity of inflammatory activation during STEMI and the development of LVSD with increased risk of HF, which indicates a need for further research to address the potential usefulness of anti-inflammatory therapies in patients with enhanced inflammation in the acute phase of STEMI for reducing the incidence of these adverse outcomes [11,27,37,44]. Previous small pilot trials of inflammatory inhibition with anakinra, a human recombinant IL-1 receptor agonist, in clinically stable patients with acute STEMI and near-normal LV dimensions and function indicated that treatment with anakinra may prevent new-onset HF in short- and long-term observation. However, they were inconclusive in terms of its impact on LV systolic function [45,46,47]. Given the results of the CANTOS study and a few pilot trials, if confirmed in larger trials, IL-1 blockade during STEMI may represent a promising avenue for research on therapeutic strategies to prevent LVSD and HF [39,40,42,45,46,47,48]. Notwithstanding these previous results, IL-1 inhibition and other anti-inflammatory treatments targeting different pathways of inflammatory activation during the acute phase of STEMI to reduce the risk of these outcomes deserve further large prospective studies involving long-term standardized imaging of LV function and well-defined clinical endpoints. In addition, there is a need for additional studies to assess optimal duration, dose, and timing of anti-inflammatory treatment to achieve sufficient and durable suppression of inflammation following discontinuation of the drug, while maintaining the optimal safety profile [27,37,39,40,42,45,46,47,48,49]. In the overall context of potential anti-inflammatory treatment, our findings point to a particular need for the early identification of high-risk patients with enhanced inflammation in the acute phase of STEMI as manifested by elevated CRP concentration, which may be critical to provide pathophysiologic guidance for the early implementation of personalized treatment approaches modifying inflammatory response.

Biomarkers offer a desirable strategy for improving risk stratification and clinical decision making for STEMI patient management [7,19,50]. Our findings indicate that CRP provides useful information for the prediction of risk of post-STEMI LVSD and HF hospitalization, which enhances the overall prognostic value of already well-known risk factors (e.g., elevated BNP concentration, which is related to adverse clinical outcomes including reduced LVEF and increased rate of hospitalization for HF) [7,19,50,51]. Other biomarkers may also be potentially valuable for monitoring and predicting of HF development, for example ST2 protein [52].

The sample size of LVSD_6M_ and HF hospitalization group was insufficient for multivariable analysis to predict HF hospitalization. Prior treatment with statins may be a limiting factor by influencing CRP levels, but this is relevant to only <10% of patients, and CRP, cholesterol levels at admission did not differ between patients with or without LVSD_6M_. The possibility that higher HF hospitalization rate in patients with LVSD_6M_ might be influenced by greater prevalence of mild HF prior to STEMI is weakly limiting because of long-term follow-up. Potential circadian and seasonal variations in CRP concentrations or genetic variants of CRP contributing to variability in CRP concentrations were unaccounted for, which is a common limitation that is difficult to avoid.

## 4. Materials and Methods

### 4.1. Study Design

This study was performed in the Nicolaus Copernicus University in Toruń, Collegium Medicum in Bydgoszcz, Poland in accordance with the Declaration of Helsinki. Approval from the Bioethics Committee of the Collegium Medicum, Nicolaus Copernicus University (KB 440/2004) was obtained (5 October 2004). All patients provided informed consent. This was a single-center prospective cohort study with rigorous selection criteria, adequate sample size, and long-term follow-up data based on multiple assessment time points: baseline, 24 h and discharge during index hospitalization for STEMI, 1 month and 6 months after discharge, and long-term follow-up. Serial measurements at multiple time points of high-sensitivity CRP concentration were made to determine the optimal time point that is the strongest predictor of clinical outcomes. Neurohormonal activation and hemodynamic stress were assessed using BNP plasma concentration measurements.

Inclusion criteria in the study protocol were: (1) first STEMI, (2) typical stenocardial chest pain of ≥30 min, (3) symptoms <12 h before admission, (4) ST-segment elevation of ≥0.2 mV in V1 through V3 precordial leads or ≥0.1 mV in ≥2 other leads in electrocardiogram [45]. Exclusion criteria involved potentially confounding factors that could affect cardiac injury, cardiac function assessment [53], and inflammatory response, such as prior myocardial damage and coronary revascularization or thrombolysis, cardiogenic shock on admission, elevated creatinine concentration (>176.8 µmol/L), HF symptoms of NYHA (New York Heart Association) class ≥III, hemodynamically significant valvular heart disease, primary cardiomyopathies, atrial fibrillation, active inflammatory or neoplastic process on admission, and therapy with steroids, immunosuppressive agents, and non-steroidal anti-inflammatory drugs. These conditions were excluded before enrollment of patients to the study by careful standard clinical evaluation by the attending cardiologist.

According to the study protocol the primary and secondary endpoints were specified as follows. LVSD_6M_, defined as LVEF ≤ 40% in echocardiography at 6 months after discharge post-STEMI, was the primary study endpoint [1,2,3,4,9,10]. A need for hospitalization for HF (referred to as HF hospitalization) in patients with LVSD_6M_ in long-term follow-up was evaluated as the secondary study endpoint. HF hospitalization was defined as post-index STEMI readmission due to new or increasing symptoms and signs of HF including fluid retention or other objective evidence of HF (such as increasing dyspnea, peripheral edema, bilateral post-tussive rales in at least the lower third of lung fields, or ventricular gallop rhythm) in combination with a change in treatment to improve HF including parenteral use of diuretic [8]. HF prior to STEMI or at discharge upon index hospitalization for STEMI was defined as a syndrome in which the patients have the following features: symptoms of HF, typically shortness of breath at rest or during exertion, and/or fatigue; signs of fluid retention such as pulmonary congestion or ankle swelling; and objective evidence of an abnormality of the structure or function of the heart at rest [54]. The NYHA functional classification of HF was used to evaluate the severity of HF prior to STEMI or at discharge upon index hospitalization for STEMI.

Patients were enrolled in this study between December 2005 and December 2008. At hospital discharge after STEMI, follow-up visits at hospital clinic were scheduled 1 and 6 months after discharge. Data on death and HF hospitalization were acquired at follow-up visits (based on discharge cards collected from patients) and from the National Health Fund registry (based on the record of HF hospitalizations, specifically only the hospitalizations encoded as HF hospitalizations satisfying the above-given criteria of the definition of HF hospitalization were considered) until 2012, which represents a long-term follow-up. Comparisons between subgroups of patients with or without LVSD_6M_ and subgroups with LVSD_6M_ and with or without HF hospitalization in long-term follow-up were made.

### 4.2. Therapy

All patients received standard treatment following current guidelines on the management of acute STEMI developed by the European Society of Cardiology [35,36]. Immediately after a diagnosis of STEMI, all patients received oral loading doses of clopidogrel (600 mg) and aspirin (300 mg), as well as intravenously unfractionated heparin (70 IU/kg, up to 5000 IU). All patients upon arrival to the hospital were transmitted directly to the cardiac catheterization lab to perform prompt coronary angiography. In the catheterization laboratory, a second dose of unfractionated heparin was administered intra-arterially in a weight-adjusted manner (up to 100 IU/kg) or under activated clotting time guidance (to the target range of 200–250 s) when abciximab was intended. Coronary angiography and primary PCI were performed by an experienced interventional cardiologist according to standard techniques using the femoral approach. The operator was blinded to the study protocol. Coronary stenting of culprit coronary vessel lesion was the technique of choice for all admitted patients. Intracoronary stents were routinely implanted. The use of abciximab and aspiration thrombectomy were done at the operator’s discretion. Thrombolysis in Myocardial Infarction (TIMI) 3 flow and TIMI Myocardial Perfusion Grade (TMPG) 3 were used to define reperfusion success [55]. The analyses of all angiographic data were performed offline by two observers blinded to echocardiographic data, clinical outcomes, and biomarker levels.

Clopidogrel up to 1 year and aspirin, each at 75 mg q.d., were continued in all patients except for patients with allergy to aspirin. Concomitant medications included perindopril and long-acting metoprolol in doses adjusted for resting heart rate and blood pressure in all patients who tolerate these medications and without contraindications, regardless of blood pressure or LV function, and simvastatin at 40 mg q.d. regardless of cholesterol level to achieve low-density lipoprotein cholesterol <80 mg/dL in all patients in the absence of contraindications. This treatment was administered in the acute phase of STEMI and was continued long-term after STEMI. We monitored that these medications were continued throughout the study period for at least 12 months after hospital discharge post-STEMI and made recommendations for continued guideline-based pharmacotherapy beyond this 12-month period. Other medications such as spironolactone or non-potassium-sparing diuretics were administered depending on the indications.

### 4.3. Echocardiography

Two-dimensional transthoracic echocardiography employing the Doppler technique (SONOS 7500 Ultrasound System, Philips, Bothell, WA, USA) was performed at discharge and after 6 months following the American Society of Echocardiography recommendations [56,57]. Echocardiographic recordings were assessed offline by two independent experienced experts blinded to the time point, clinical outcomes, and biomarker levels. Measurements from three consecutive cardiac cycles were averaged. The echocardiographic results obtained by both experts were averaged. LV volumes and LVEF were calculated using the biplane method of discs (modified Simpson’s rule) in two- and four-chamber views, which is the recommended method of choice for LVEF assessment in echocardiography [56,57]. The principle underlying this method is that the total LV volume is calculated from the summation of a stack of elliptical discs. The height of each disc is calculated as a fraction of the LV long axis based on the longer of the two lengths from the two- and four-chamber views. The cross-sectional area of the disk is based on the two diameters obtained from the two- and four-chamber views. The inter- and intra-observer coefficients of variation (CVs) for LVEF in the first 50 patients were below 5% and 2.5%, respectively.

We note that while we used two-dimensional echocardiography for obtaining LVEF (which is a recommended method for LV systolic function assessment in STEMI patients) [10,35,36], there are also other imaging techniques for assessing LV function [58].

### 4.4. Blood Sampling and Biomarkers

Peripheral venous blood samples were collected using ethylenediaminetetraacetic acid tubes. After centrifugation, the plasma was stored at −80 °C until analysis. High-sensitivity CRP plasma concentration was measured with an ultra-sensitive latex immunoassay (CRP Vario test, analyzer ARCHITECT ci 8200, Abbott, Abbott Park, IL, USA) at admission (CRP_AD_), 24 h after admission (CRP_24_), at discharge (CRP_DC_), and 1 month after discharge (CRP_1M_). BNP concentration was measured with a chemiluminescent microparticle immunoassay (ARCHITECT ci 8200) at admission and discharge. Detection limit for CRP was 0.1 mg/L and for BNP 10 pg/L. Intra-assay CV was <2% for CRP and <5% for BNP, and for inter-assay <1% and <5%, respectively. CRP_1M_ ≥ 2 mg/L was used as a criterion of persistent pro-inflammatory response.

### 4.5. Statistics

The Shapiro–Wilk test demonstrated a lack of normal distribution for the majority of the investigated variables. Continuous variables are reported as median values and interquartile ranges. Depending on the presence or absence of a normal distribution, inter-group comparisons were performed with Student’s *t*-tests for independent samples or Mann–Whitney unpaired rank sum test. Intra-group comparisons were made with Student’s *t*-tests for paired samples or Wilcoxon matched-paired rank sum tests. Categorical variables were compared using the χ2 test with the Yates correction. Univariate and multivariable logistic regression models were used to identify markers of LVSD_6M_. The first basic multivariable model was based on generalized linear/nonlinear model (GLZ). Given that the strong prognostic significance of depressed LVEF at discharge has long been proven, we expected that this first basic model may not reveal the potential prognostic value of other factors, especially high-sensitivity CRP during an index STEMI. Therefore, a second multivariable model was examined to investigate if other markers including high-sensitivity CRP provide an added predictive value for LVSD_6M_. In this second model LVEF at discharge was purposely excluded. Relations between investigated variables and likelihood of LVSD_6M_ were estimated with odds ratios and 95% confidence intervals. Receiver-operator-characteristic analysis was made to determine diagnostic accuracy of LVSD_6M_ prediction and optimal cut-off points. Kaplan–Meier method was used to estimate the probability of event-free survival. A two-sided *p*-value of <0.05 was considered significant. For estimating the final sample size, we performed an internal pilot study of the first 50 patients in whom CRP concentrations and LVEF were measured. Based on these results and assuming a two-sided α of 0.05, we calculated using the *t*-test for independent variables that enrollment of 200 patients would provide a 99.9% and 98.9% power to demonstrate significant differences in CRP_24_ and CRP_DC_, respectively, between patients with and without LVSD_6M_. Statistical analysis was made using Statistica v. 12.0 (StatSoft, Tulsa, OK, USA) and MedCalc v. 12.0 (MedCalc Software, Mariakerke, Belgium). 

## 5. Conclusions

The risk of long-term post-infarct LVSD and HF was increased in patients with enhanced inflammation during STEMI, as manifested by elevated high-sensitivity CRP concentration. CRP_24_ ≥ 19.67 mg/L, especially with persistent CRP_1M_ ≥ 2 mg/L, aided in identifying patients at high risk. The progression of LVSD and development of HF in long-term follow-up post-STEMI could be more accurately predicted owing to the incorporation of CRP as a synergistic marker into risk stratification. This strategy could improve clinical decision making for post-STEMI management including aggressive and personalized treatment approaches to prevent long-term LVSD and HF.

## Figures and Tables

**Table 1:** Baseline clinical characteristics of the whole study group of patients with ST-segment elevation myocardial infarction and two patient subgroups which were found with or without left ventricular systolic dysfunction 6 months after discharge.

Variable	Whole Study Group (*n* = 199)	Patients with LVSD 6 Months after Discharge (*n* = 35)	Patients without LVSD 6 Months after Discharge (*n* = 164)	*p*-Value between Patients with or without LVSD
Age (years)	56.0 (50.0–64.0)	58.0 (52.0–66.0)	56.0 (50.0–64.0)	0.233
Gender (male:female) *n* (%)	154:45 (77.4:22.6)	26:9 (74.3:25.7)	128:36 (78.1:21.9)	0.633
Anterior STEMI *n* (%)	88 (44.2)	31 (88.6)	57 (34.8)	<0.001
HF prior to STEMI (≤II NYHA) *n* (%)	7 (3.5)	3 (8.6)	4 (2.4)	0.009
HF at discharge (≥II NYHA) *n* (%)	20 (10.1)	10 (28.6)	10 (6.1)	<0.001
Body mass index (kg/m²)	26.7 (24.2–29.1)	27.9 (26.0–30.1)	26.2 (24.0–29.0)	0.075
Hypertension *n* (%)	82 (41.2)	19 (54.3)	63 (38.4)	0.083
Diabetes mellitus *n* (%)	35 (17.6)	10 (27.0)	25 (15.4)	0.095
Long-acting metoprolol at discharge *n* (%)	197 (99.0)	35 (100.0)	162 (98.8)	0.782
Perindopril at discharge *n* (%)	198 (99.5)	35 (100.0)	163 (99.4)	0.393
Simvastatin at discharge *n* (%)	199 (100.0)	35 (100.0)	164 (100.0)	1.0
Spironolactone at discharge *n* (%)	15 (7.5)	9 (25.7)	6 (3.7)	<0.001
Non-potassium sparing diuretics at discharge *n* (%)	12 (6.0)	7 (20.0)	5 (3.1)	<0.001
Creatinine at admission (µmol/L)	83.1 (72.5–97.2)	88.4 (79.6–97.2)	79.6 (71.6–97.2)	0.152
Glucose at admission (mmol/L)	7.6 (6.8–9.3)	8.61 (7.17–10.7)	7.31 (6.67–9.19)	0.004
Leukocyte count at admission (10³/µL)	11.2 (9.1–13.2)	11.7 (10.6–13.4)	10.9 (8.76–13.1)	0.312
Leukocyte count 24 h after admission (10³/µL)	10.1 (5.4–20.1)	12.3 (8.84–13.6)	10.1 (8.28–11.2)	<0.001
LDL cholesterol at admission (mmol/L)	3.8 (3.2–4.4)	3.72 (3.34–4.45)	3.78 (3.21–4.45)	0.452
CK-MB_max_ (U/L)	102.5 (57.5–159.5)	178.0 (122.0–231.0)	94.0 (54.0–137.0)	<0.001
TnI_max_ (ng/mL)	44.2 (11.3–>50.0)	>50.0 (>50.0–>50.0)	33.9 (10.1–>50.0)	<0.001
CRP at admission (mg/L)	1.74 (0.98–3.29)	1.72 (1.12–3.06)	1.79 (0.945–3.33)	0.703
CRP 24 h after admission (mg/L)	10.13 (5.62–19.64)	22.6 (12.1–33.9)	9.06 (5.36–15.5)	<0.001
CRP at discharge (mg/L)	10.09 (4.9–17.65)	17.9 (8.09–35.6)	9.02 (4.47–16.2)	<0.001
CRP 1 month after discharge (mg/L)	1.7 (0.87–3.27)	2.12 (1.01–3.17)	1.51 (0.830–3.21)	0.833
BNP at admission (pg/mL)	51.9 (5.7–101.4)	74.6 (29.1–156.9)	50.9 (25.5–89.9)	0.001
BNP at discharge (pg/mL)	123.0 (70.4–226.9)	336.5 (227.2–717.5)	106.7 (62.2–169.2)	<0.001

Data represent median values with corresponding interquartile range (in parentheses). Abbreviations: BNP—B-type natriuretic peptide; CK-MB_max_—maximal activity of creatine kinase-MB; CRP—high-sensitivity C-reactive protein; HF—heart failure; LDL cholesterol—low-density lipoprotein cholesterol; LVSD—left ventricular systolic dysfunction; NYHA—New York Heart Association classification; STEMI—ST-segment elevation myocardial infarction; TnI_max_—maximal concentration of troponin I.

**Table 2:** Angiographic and echocardiographic characteristics of the whole study group of patients with ST-segment elevation myocardial infarction and two patient subgroups, which were found with or without left ventricular systolic dysfunction 6 months after discharge.

Variable	Whole Study Group (*n* = 199)	Patients with LVSD 6 Months after Discharge (*n* = 35)	Patients without LVSD 6 Months after Discharge (*n* = 164)	*p*-Value between Patients with or without LVSD
**Angiographic indices:**				
LAD/non-LAD *n* (%)	92 (46.2)/107 (53.8)	32 (91.4)/3 (8.6)	60 (36.6)/104 (63.4)	<0.001
TIMI 3 flow pre-PCI *n* (%)	55 (27.6)	3 (8.6)	52 (31.7)	0.035
TIMI 3 flow post-PCI *n* (%)	185 (93.0)	29 (82.9)	156 (95.1)	0.02
TMPG 3 post-PCI *n* (%)	92 (46.2)	15 (42.9)	77 (47.0)	0.659
Multivessel coronary disease *n* (%)	119 (59.8)	22 (62.9)	97 (59.1)	0.638
Abciximab use *n* (%)	50 (25.1)	15 (42.9)	35 (21.3)	0.007
Intracoronary stents *n* (%)	197 (99.0)	35 (100.0)	162 (98.8)	0.197
**Echocardiographic indices at discharge:**				
LVEDd (mm)	49.0 (45.0–53.0)	53.0 (52.0–57.0)	48.0 (45.0–52.0)	<0.001
LVESd (mm)	34.0 (30.0–37.0)	38.0 (36.0–45.0)	33.0 (30.0–35.0)	<0.001
LVEDVI (mL/m²)	48.4 (44.2–60.6)	60.4 (53.9–69.6)	48.6 (43.0–57.9)	<0.001
LVESVI (mL/m²)	26.8 (23.1–34.9)	37.7 (33.4–43.8)	25.8 (22.9–30.7)	<0.001
LVEF (%)	45.0 (40.0–49.7)	36.0 (33.5–40.0)	46.0 (42.5–50.0)	<0.001
WMSI (pts)	1.5 (1.38–1.75)	1.81 (1.75–1.94)	1.44 (1.38–1.69)	<0.001
S′ (cm/s)	7.0 (6.1–8.1)	5.9 (5.1–6.8)	7.2 (6.4–8.4)	<0.001
DT (ms)	155.0 (145.0–185.0)	145.0 (135.0–155.0)	160.0 (150.0–190.0)	<0.001
E/E′ (−)	10.3 (8.4–12.6)	11.8 (9.6–13.9)	10.1 (8.2–12.0)	0.005
**Echocardiographic indices 6 months after discharge:**				
LVEDd (mm)	50.0 (46.0–54.0)	55.0 (50.0–56.0)	48.5 (45.5–53.0)	<0.001
LVESd (mm)	34.0 (31.0–37.0)	39.0 (35.0–44.0)	33.0 (31.0–36.0)	<0.001
LVEDVI (mL/m²)	57.4 (48.8–68.6)	76.9 (68.1–83.1)	54.5 (43.2–65.0)	<0.001
LVESVI (mL/m²)	29.3 (24.8–39.0)	48.2 (40.9–56.4)	27.7 (24.1–33.6)	<0.001
LVEF (%)	46.0 (42.3–52.0)	36.0 (34.0–38.6)	47.7 (44.2–52.5)	<0.001
WMSI (pts)	1.44 (1.31–1.63)	1.88 (1.69–1.94)	1.38 (1.31–1.5)	<0.001
S′ (cm/s)	7.0 (6.1–8.1)	5.6 (4.8–6.9)	7.1 (6.3–8.2)	<0.001
DT (ms)	170.0 (155.0–195.0)	150.0 (135.0–190.0)	175.0 (155.0–200.0)	<0.001
E/E′ (−)	9.5 (8.0–11.7)	13.1 (9.7–16.3)	9.1 (7.9–10.8)	<0.001

Data represent median values with corresponding interquartile range (in parentheses). Abbreviations: DT—deceleration time of early transmitral flow; E—peak velocity of early transmitral flow; E′—average peak early diastolic mitral annular velocity; LAD—infarct-related left descending artery; LVEDd—left ventricular end-diastolic diameter; LVEDVI—left ventricular end-diastolic volume index; LVEF—left ventricular ejection fraction; LVESd—left ventricular end-systolic diameter; LVESVI—left ventricular end-systolic volume index; LVSD—left ventricular systolic dysfunction; PCI—primary percutaneous coronary intervention; S′—average peak systolic mitral annular velocity; STEMI—ST-segment elevation myocardial infarction; TIMI—Thrombolysis in Myocardial Infarction score; TMPG—TIMI Myocardial Perfusion Grade; WMSI—wall motion score index.

**Table 3:** Baseline characteristics of two patient groups with left ventricular systolic dysfunction 6 months after discharge post ST-segment elevation myocardial infarction based on the need for hospitalization for heart failure in long-term follow-up.

Variable	With HF Hospitalization in Long-Term Follow-Up (*n* = 16)	Without HF Hospitalization in Long-Term Follow-Up (*n* = 19)	*p*-Value
Age (years)	57.0 (53.0–64.0)	61.0 (50.0–67.0)	0.935
Gender (male:female) *n* (%)	11:5 (68.8:31.3)	15:4 (78.9:21.1)	0.492
Anterior location of STEMI *n* (%)	15 (93.8)	16 (84.2)	0.365
HF prior to STEMI (I/II NYHA) *n* (%)	1 (6.3)	2 (10.5)	0.082
HF at discharge for STEMI (≥II NYHA) *n* (%)	5 (31.3)	5 (26.3)	0.418
Body mass index (kg/m²)	29.4 (27.3–30.7)	27.4 (24.5–29.4)	0.088
Hypertension *n* (%)	8 (50)	11 (57.9)	0.640
Diabetes mellitus *n* (%)	5 (31.3)	5 (26.3)	0.748
Creatinine at admission (µmol/L)	88.4 (79.6–98.1)	88.4 (70.7–91.3)	0.656
Glucose at admission (mmol/L)	7.75 (6.75–10.8)	9.00 (7.94–10.7)	0.125
Leukocyte count on admission (10³/µL)	12.6 (10.9–13.6)	11.7 (9.50–12.5)	0.441
Leukocyte count 24 h after admission (10³/µL)	12.4 (9.38–14.0)	12.3 (8.74–13.4)	0.461
LDL cholesterol at admission (mmol/L)	3.63 (3.26–5.01)	3.72 (3.44–4.22)	0.781
CK-MB_max_ (U/L)	203.5 (157.5–240.0)	148.0 (119.0–206.0)	0.172
TnI_max_ (ng/mL)	>50.0 (>50.0–>50.0)	>50.0 (>50.0–>50.0)	0.350
CRP at admission (mg/L)	2.59 (1.42–4.24)	1.6 (0.82–1.77)	0.014
CRP 24 h after admission (mg/L)	29.5 (17.8–43.8)	20.11 (6.21–26.7)	0.056
CRP at discharge (mg/L)	21.9 (12.36–43.7)	15.4 (7.95–24.66)	0.161
CRP 1 month after discharge (mg/L)	2.57 (1.69–3.48)	1.54 (0.65–2.72)	0.052
BNP at admission (pg/mL)	108.9 (23.2–269.7)	61.9 (31.0–132.4)	0.502
BNP at discharge (pg/mL)	384.8 (198.8–756.0)	336.5 (233.0–717.5)	0.781
LAD/non-LAD n (%)	15 (93.8)	17 (89.8)	0.649
TIMI 3 flow pre-PCI *n* (%)	2 (12.5)	1 (5.3)	0.733
TIMI 3 flow post-PCI *n* (%)	14 (87.5)	15 (78.9)	0.978
TMPG 3 post-PCI *n* (%)	7 (43.8)	8 (42.1)	0.615
Multivessel coronary disease *n* (%)	11 (68.8)	11 (57.9)	0.507
Abciximab use *n* (%)	7 (43.8)	8 (42.1)	0.922
LVEF at discharge for STEMI (%)	36.0 (32.8–39.7)	36.8 (35.0–40.4)	0.301
WMSI at discharge for STEMI (pts)	1.88 (1.81–1.97)	1.81 (1.75–1.88)	0.095
LVEF 6 months after discharge for STEMI (%)	35.8 (31.3–37.0)	36.9 (35.0–39.2)	0.056
WMSI 6 months after discharge for STEMI (pts)	1.88 (1.84–1.97)	1.75 (1.63–1.88)	0.029

Data represent median values with corresponding interquartile range (in parentheses). Abbreviations: BNP—B-type natriuretic peptide; CK-MB_max_—maximal activity of isoenzyme MB of creatine kinase; CRP—high-sensitivity C-reactive protein; HF—heart failure; HDL cholesterol—high-density lipoprotein cholesterol; LAD—infarct-related left descending artery; LDL cholesterol—low-density lipoprotein cholesterol; LVEF—left ventricular ejection fraction; LVSD—left ventricular systolic dysfunction; NYHA—New York Heart Association; PCI—primary percutaneous coronary intervention; STEMI—ST-segment elevation myocardial infarction; TIMI—Thrombolysis in Myocardial Infarction score; TMPG—TIMI Myocardial Perfusion Grade; TnI_max_—maximal concentration of troponin I; WMSI—wall motion score index.

**Table 4:** Predictors of post-infarct left ventricular systolic dysfunction in a univariate and multivariable analysis.

Variable	OR	95% CI	*p*-Value
**Univariate analysis:**			
WMSI at discharge (for a 1-point increase)	8255.0	387.65–175,791.3	<0.001
LAD vs. non-LAD	17.10	4.99–58.62	<0.00001
Anterior vs. nonarterior wall STEMI	14.54	4.86–43.55	<0.000003
Abciximab use vs. no use	2.80	1.29–6.07	<0.01
BNP at discharge (for a 100 pg/mL increase)	1.81	1.44–2.28	<0.000001
CRP at 24 h after admission (for a 10 mg/L increase)	1.62	1.29–2.03	<0.00006
BNP at admission (for a 100 pg/mL increase)	1.48	1.06–2.05	<0.03
CRP at discharge (for a 10 mg/L increase)	1.45	1.17–1.80	<0.0008
WBC 24 h after admission (for a 10³/µL increase)	1.27	1.10–1.46	<0.001
LVESd at discharge (for a 1 mm increase)	1.22	1.12–1.33	<0.00001
LVEDd at discharge (for a 1 mm increase)	1.16	1.08–1.26	<0.0002
LVESVI at discharge (for a 1 mL/m² increase)	1.15	1.09–1.20	<0.0000003
E/E’ at discharge (for a 1-point increase)	1.13	1.03–1.24	<0.01
CK-MB_max_ (for a 10 U/L increase)	1.10	1.05–1.15	<0.00006
Body mass index (for a 10 kg/m² increase)	1.09	0.99–1.20	0.08
LVEDVI at discharge (for a 1 mL/m² increase)	1.07	1.04–1.10	<0.00003
TnI_max_ (for a 1 ng/mL increase)	1.06	1.03–1.10	<0.0003
WBC at admission (for a 10³/µL increase)	1.06	0.94–1.20	0.313
DT at discharge (for a 1 ms increase)	0.97	0.96–0.99	<0.002
CRP at admission (for a 10 mg/L increase)	0.96	0.09–5.19	0.698
LVEF at discharge (for a 1% increase)	0.70	0.62–0.79	<0.0000002
TIMI flow pre-PCI (for a 1-point increase)	0.63	0.44–0.88	<0.008
S’ at discharge (for a 1 cm/s increase)	0.45	0.32–0.64	<0.00002
**Multivariable analysis (MA):**			
BNP at discharge (for a 100 pg/mL increase)	1.44	1.11–1.84	<0.0002
LVEF at discharge (for a 1% increase)	0.73	0.65–0.83	<0.00002
**MA with LVEF at discharge excluded:**			
LAD vs. non-LAD	7.36	1.95–27.7	<0.004
BNP at discharge (for a 100 pg/mL increase)	1.59	1.26–2.01	<0.0002
CRP at 24 h after admission (for a 10 mg/L increase)	1.47	1.10–1.97	<0.01

Univariate analysis shows parameters from Table 1 and Table 2 with a *p*-value < 0.01, as well as high-sensitivity C-reactive protein and leukocyte count independent of a *p*-value. Results are presented according to decreasing values of odds ratios. Abbreviations: BNP—B-type natriuretic peptide; CI—confidence interval; CK-MB_max_—maximal activity of isoenzyme MB of creatine kinase; CRP—high-sensitivity C-reactive protein; DT—deceleration time of early transmitral flow; E—peak velocity of early transmitral flow; E’—average peak early diastolic mitral annular velocity; LAD—infarct-related left descending artery; LVEDd—left ventricular end-diastolic diameter; LVEDVI—left ventricular end-diastolic volume index; LVEF—left ventricular ejection fraction; LVESd—left ventricular end-systolic diameter; LVESVI—left ventricular end-systolic volume index; OR—odds ratio; S’—average peak systolic mitral annular velocity; STEMI—ST-segment elevation myocardial infarction; TIMI—Thrombolysis in Myocardial Infarction score; TnI_max_—maximal concentration of troponin I; WBC -leukocyte count; WMSI—wall motion score index.

## Abbreviations

BNP	B-type natriuretic peptide
CRP	C-reactive protein
CRP_AD_	C-reactive protein concentration at hospital admission
CRP_24_	C-reactive protein concentration 24 h after hospital admission
CRP_DC_	C-reactive protein concentration at hospital discharge
CRP_1M_	C-reactive protein concentration 1 month after hospital discharge
HF	heart failure
IL	interleukin
LVEF	left ventricular ejection fraction
LVSD	left ventricular systolic dysfunction
LVSD_DC_	left ventricular systolic dysfunction at hospital discharge
LVSD_6M_	left ventricular systolic dysfunction 6 months after hospital discharge
NYHA	New York Heart Association
PCI	percutaneous coronary intervention
STEMI	ST-segment elevation myocardial infarction
TIMI	Thrombolysis in Myocardial Infarction
TMPG	TIMI Myocardial Perfusion Grade
